# 763. Infection Prevention Resource Physicians to Build On-Call Capacity

**DOI:** 10.1093/ofid/ofad500.824

**Published:** 2023-11-27

**Authors:** Ericka Hayes, Sarah Smathers, Sara Townsend, Lauren Le Goff, Marisse Plaras, Audrey John, Yasaman Fatemi, Julia Sammons

**Affiliations:** Children's Hospital of Philadelphia, Philadelphia, Pennsylvania; Children's Hospital of Philadelphia, Philadelphia, Pennsylvania; Children's Hospital of Philadelphia, Philadelphia, Pennsylvania; Children's Hospital of Philadelphia, Philadelphia, Pennsylvania; Children's Hospital of Philadelphia, Philadelphia, Pennsylvania; Children's Hospital of Philadelphia, Philadelphia, Pennsylvania; Seattle Children's Hospital/University of Washington, Seattle, Washington; Children's Hospital of Philadelphia, Philadelphia, Pennsylvania

## Abstract

**Background:**

The COVID-19 pandemic showed that infection prevention and control (IPC) human resources are critical and are stretched in prolonged crisis. There has also been a shift from experienced infection preventionists (IPs) during the pandemic and natural attrition to a less experienced cohort. Medical Director (MD) oversight of IPC programs is critical in providing clinical guidance and support for developing IPs. IPC and MD resources are required 24/7 to respond to urgent issues such as management of highly infectious exposures. Many IPC programs have a single MD with extensive on call periods stretching a limited resource and increasing potential for physician burnout. We report the impact of Infectious Diseases Resource Physicians (IDRP) to support IPC on call.

**Methods:**

Our large pediatric health system had a gap in MD coverage from June 2022-October 2022. To support IPC on call, we developed a weekly rotating IDRP staffed by an inpatient ID physician with MD backup as needed. After a senior MD onboarded in October 2022, the IDRP rotation was expanded to other ID physicians on a voluntary basis. Education in management of common urgent/emergent IPC issues was provided to all IDRPs. Data was collected on time spent and themes by IDRPs.

**Results:**

From October 2022-April 2023 we have had 11 IDRPs provide IPC support. Calls per week ranged from 0-5 with a median of 1. Time investment by IDRP weekly was a median of 10 minutes, range 0-60 minutes, spent supporting IPC questions. Common themes were communicable diseases such as tuberculosis, assessing symptomatic caregivers/parents and type of precautions needed. Feedback from the IPC team and IDRPs was positive as solicited through meetings, emails and individual inquiry.

**Conclusion:**

We have successfully established ID physician support coverage for our IPs on call, building a bench of IPC expertise throughout our ID group and supporting the single senior MD. Additional capacity for the IPC program provides flexibility and support needed during critical times, such as pandemic response. IPs felt well supported by the IDRP with the additional benefit of strengthening relationships across the IPC team and ID division. Key factors in success include clear expectations, education, feedback, and support.

**Disclosures:**

**All Authors**: No reported disclosures

